# Supplementary material for: Peritoneal Fluid Analysis of Advanced Ovarian Cancers after Hyperthermic Intraperitoneal Chemotherapy
Source: Int J Mol Sci. 2023 Jun 5;24(11):9748. doi: 10.3390/ijms24119748 (PMC10253890; doi:10.3390/ijms24119748)

Table S1. The levels of cytokines with significantly differential expression at different timings

	Pre-HIPEC	Post-HIPEC	24 hrs	48 hrs	72 hrs	P-value (Pre vs. post HIPEC)
RANTES	332	2131	84.5	72	68	0.0001
G-CSF	7	331.5	546.5	55	23.5	0.0108
MCP-1	1.5	257.5	319	321	338.5	0.0112
TIMP-1	45170	138066	90948.5	62130.5	56701	0.025
IL-6sR	824	1194.5	946.5	1433.5	1555	0.0291
IL-6	13.5	1964	1062	668	237.5	0.0309

Figure S1. Enrichment map of the top 10 biological process (BP) Enrichment GO terms. The map covered the pathways involved by the genes regulated by the miRNAs with significantly differential expression. The pathways with red color indicate the much closer relationship among the related genes.

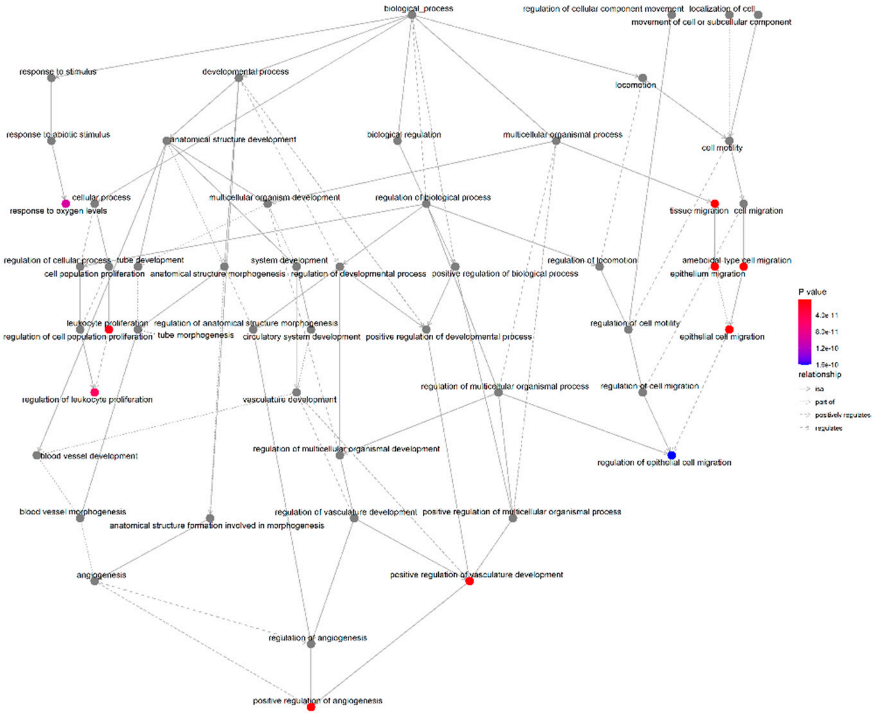

Supplement: Supplementary file 1 [file supplementary materials.pdf › supplementary materials.pdf]
